# Molecular phenotypes associated with anomalous stamen development in *Alternanthera philoxeroides*

**DOI:** 10.3389/fpls.2015.00242

**Published:** 2015-04-14

**Authors:** Zhu Zhu, Chengchuan Zhou, Ji Yang

**Affiliations:** ^1^Ministry of Education Key Laboratory for Biodiversity Science and Ecological Engineering, Center for Evolutionary Biology, School of Life Sciences, Fudan UniversityShanghai, China; ^2^Shanghai Key Laboratory of Plant Functional Genomics and Resources, Shanghai Chenshan Botanical GardenShanghai, China

**Keywords:** *Alternanthera philoxeroide*, sexual dysfunction, aberrant stamen development, male sterility, molecular phenotypes

## Abstract

*Alternanthera philoxeroides* is a perennial amphibious weed native to South America but has now spread to diverse parts of the world. *A. philoxeroides* reproduces both sexually and asexually in its native range, but propagates solely through vegetative means in its introduced range. Traits associated with sexual reproduction become degraded for sexual dysfunction, with flowers possessing either pistillate stamens or male-sterile anthers. Degradations of sexual characters for loss of sexuality commonly take place in clonal plants. The underlying molecular-genetic processes remain largely unknown. We compared the gene expression profiles of abnormal stamens with that of normal stamens by RNA-Seq analysis, and identified a large number of differentially expressed genes between abnormal and normal stamens. In accordance with flower morphology, the expression of B-class MADS-box genes (*ApAP3, ApTM6,* and *ApPI*) was markedly reduced in pistillate stamens. However, most of the genes involved in meiosis were expressed normally in stamens with male-sterile anthers. In addition to verifying the expression patterns of genes previously known to be related to stamen and pollen grain development, we also identified previously unknown molecular phenotypes associated with sexual dysfunction in *A. philoxeroides*, that is helpful for dissecting the molecular mechanisms underpinning various male-sterile phenotypes and the molecular processes underlying the transition from sexuality to asexuality in clonal plants.

## Introduction

*Alternanthera philoxeroides*, commonly known as alligator weed, is a perennial amphibious weed native to South America, but has now spread to diverse parts of the world, showing up in North and South America, France, Italy, Australia, New Zealand, China, and other parts of Asia. *A. philoxeroides* can grow in a variety of habitats, including open lands, waterway banks, ponds, and lakes. Individuals growing in aquatic and terrestrial habitats showed extensive variations in leaf size and shape, stem diameter and internode length, but exhibited little genetic differentiation within and among populations (Ye et al., 2003; Geng et al., 2007). It has thus been proposed that phenotypic plasticity, rather than locally adapted ecotypes, allows *A. philoxeroides* to colonize a wide range of habitats (Geng et al., 2006, 2007; Li and Ye, 2006).

**FIGURE 1:**
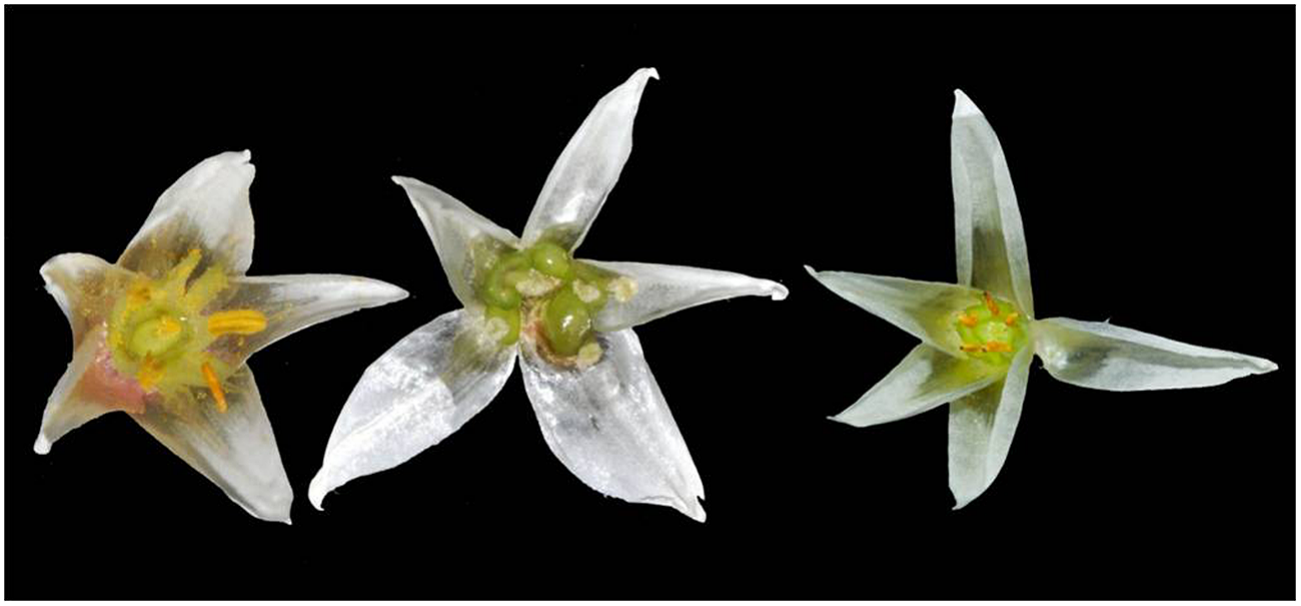
**Images of representative* Alternanthera philoxeroides* flowers: normal flowers **(left)**, pistillate flowers **(middle)**, and male-sterile flowers (right)**.

*Alternanthera philoxeroides* reproduces both sexually and asexually in its native range, but propagates mainly through vegetative means via storage root and stem fragmentation in its introduced range and does not produce viable seeds (Julien and Stanley, 1999; Sosa et al., 2007). Extensive field survey of the introduced *A. philoxeroides* in China revealed various patterns of anomalous floral development (Chen, 1964). The most striking aberration is the homeotic transformation of stamens into pistils or pistil-like structures (**Figure 1**). The complete pistillate flowers do not have stamens but five pistil-like structures and one normal pistil. Unlike the normal pistil, the ‘pistils’ transformed from stamens often develop ovary-like structures but contain no ovule inside (Chen, 1964; Hu et al., 2011). Monoclinous flowers possessing both stamens and pistils are common in natural populations. However, the anthers of these flowers are often shriveled bearing no or few non-viable pollen grains (Hu et al., 2011; Wang et al., 2011). There also exist some incomplete pistillate flowers with intermediate phenotypes between monoclinous and complete pistillate plants.

The ABC(DE) model is now widely used as a framework for understanding the molecular mechanisms controlling floral organ identity (Coen and Meyerowitz, 1991; Theißen, 2001). According to the model, the differentiation of floral organs is controlled by the differential expression of several subsets of homeotic genes belonging to the MADS-box gene family, except for the A-class gene *APETALA2* (Riechmann and Meyerowitz, 1997; Vandenbussche et al., 2003). The co-expression of B- and C-class MADS-box genes establishes the identity of the stamens (Coen and Meyerowitz, 1991; Jack et al., 1992). B-function mutants produce homeotic transformation of stamens into carpels (Jack et al., 1992; Goto and Meyerowitz, 1994). It is unclear whether pistillody in *A. philoxeroides* was caused by altering the expression pattern of the B-class MADS-box genes. Additionally, the cytotype of *A. philoxeroides* found in China is a hexaploid (Cai et al., 2009). Sosa et al. (2007) suggested a hybrid origin of the invasive hexaploid of *A. philoxeroides*, and that meiotic abnormalities due to the formation of univalents/multivalents led to microspore degeneration which resulted in anthers bearing no pollen grains. Hu et al. (2011) found, however, that the microspore tetrads were formed and separated normally in the anthers of the hexaploid *A. philoxeroides*, but the protoplasm of most pollen grains disintegrated at the post-maturation stage and pollen grains became empty, with only a few non-viable pollen grains left in the anthers.

Male sterility in plants has received considerable attention because of its potential value in breeding and hybrid seed production. It is also of great importance in evolutionary studies on the origin of dioecy (Sawhney and Shukla, 1994; Singh et al., 2012). The phenotypic manifestations of male sterility are diverse in plants, including the complete absence of male organs, the failure to develop normal sporogenous tissues (no meiosis), the abortion of pollen at any step of its development, and the inability of mature pollen to germinate on compatible stigma (Budar and Pelletier, 2001; Singh et al., 2012). The conversion of stamens to different type of floral organs also represents a male-sterile condition (Sawhney and Shukla, 1994). Although multiple genes and proteins related to microspore and pollen abortion have been characterized (Yang et al., 2003a; Jung et al., 2006; de Azevedo Souza et al., 2009), the genetic and molecular mechanisms underpinning various male-sterile phenotypes are still poorly understood. Furthermore, loss of sexuality is common in invasive clonal plants (Eckert and Barrett, 1993; Eckert, 2002; Barrett et al., 2008). Degeneration of sex can be caused by environmental and/or genetic factors. It is possible that the sexual infertility in sterile polyploids is due to polyploidy *per se* (Eckert, 2002). Traits associated with sexual reproduction may become degraded for sexual dysfunction, especially in plants that are sexually infertile and reproduction is solely clonal. However, there seems to have been a general lack of interest in dissecting the molecular-genetic processes associated with sexual infertility and degradation of sexual characters in clonal plants, even though they have arisen repeatedly in many groups of plants (Eckert, 2002). Investigation of the genetic architecture and molecular mechanisms underlying the transition from sexuality to asexuality in clonal plants will not only extend our understanding of the genetic control of reproductive organ development, but may also provide insights into the mechanisms and evolutionary pathways of sexual sterility in clonal plants.

Molecular phenotypes are important links between genomic information and organismic functions, fitness, and evolution (Held et al., 2014). In this study, we compared the gene expression profiles of abnormal stamens with that of normal stamens by RNA-Seq analysis. A large number of differentially expressed genes between abnormal and normal stamens were captured. The pistillate stamens exhibited a molecular phenotype distinct from that of the stamens with male-sterile anthers. In addition to verifying the expression patterns of genes previously known to be related to stamen and pollen grain development, we identified molecular phenotypes previously unknown to be associated with sexual dysfunction in *A. philoxeroides* that will be helpful in future analyses.

## Materials and Methods

### Plant Materials

Plants producing normal fertile flowers were collected from Argentina and maintained in the botanical garden of Yunnan University (E102°42^′^, N25°03^′^, Kunming, China). Pistillate flowers and male-sterile flowers were collected from plants growing in natural habitats close to the botanical garden. Flower heads containing flowers at different developmental stages were collected and preserved in RNAlater solution (Life Technologies, Gaithersburg, MD, USA). Five individuals were sampled from each type of flowers.

### RNA Extraction, cDNA Library Construction and Illumina Sequencing

RNAs of normal flowers, male-sterile flowers, and pistillate flowers were extracted using the RNeasy Plant Mini Kit (Qiagen, Valencia, CA, USA) and purified with the on-column DNase I digestion (Qiagen) following the manufacturer’s instructions. RNA quality was visually checked on a 1% agarose gel and by a Nanodrop 2000c Spectrophotometer (NanoDrop Technologies, Wilmington, DE, USA). RNA integrity was further verified by an Agilent 2100 Bioanalyzer (Agilent Technologies, Santa Clara, CA, USA).

cDNA libraries were constructed following the High-Throughput Illumina Strand-Specific RNA Sequencing Library protocol (Zhong et al., 2011). Briefly, poly A containing mRNA was purified from total RNA and then fragmented into small pieces. Double-stranded cDNA was synthesized from the fragmented cDNA, and Illumina sequencing adapters were ligated to the ends of the fragments. Libraries were sequenced using the HiSeq 2000 System (Illumina, San Diego, CA, USA).

### *De novo* Transcriptome Assembly and Gene Annotation

Raw reads generated by the sequencing machine were filtered to obtain high-quality reads. Reads containing adaptor sequences were discarded. Read with a PHRED quality score below 20 were also removed. *De novo* assembly was carried out using the Trinity software with default settings and a minimum contig length of 200 bp (Grabherr et al., 2011). Assembled contigs were used as input for a second assembly made with CAP3 (Huang and Madan, 1999). Redundancy was reduced using CD-HIT with a sequence similarity threshold of 95% (Li and Godzik, 2006). *De novo* assembled sequences were annotated using BLASTX against the *Arabidopsis thaliana* protein database^1^ (TAIR10_peptide), with an *e*-value cut-off of 10^-10^. BLAST searches against the Phytozome database^2^ were then done for unannotated sequences.

Clean reads from each sample were mapped back to the *de novo* assembled reference transcriptome. Gene expression levels were calculated from the number of uniquely aligned clean reads and then normalized into units of Reads Per Kilobase per Million reads mapped (RPKM; Mortazavi et al., 2008). Differentially expressed transcripts were detected using an False Discovery Rate (FDR) value cut-off ≤0.001 and the absolute value of log2 ratio ≥1. GO enrichment analysis for biological processes was carried out utilizing Fisher’s exact test with default parameters (*p* < 0.01) by the R package topGO (Alexa and Rahnenfuhrer, 2010). The REViGO web server^3^ was used to reduce the redundancy and visualize the overrepresented GO terms based on semantic similarity (Supek et al., 2011).

### Identification and Cloning of B-class MADS-Box Genes and Meiotic Genes in *A. philoxeroides*

Putative *A. philoxeroides AP3*, *TM6,* and *PI* sequences were used as queries to conduct BLAST searches against the NCBI databases^4^ to find homologous sequences. Multiple alignments of the retrieved sequences were constructed using ClustalW 2.0 (Larkin et al., 2007). A neighbor-joining tree was reconstructed by MEGA 6.0 (Tamura et al., 2013) using the Jones–Taylor–Thornton (JTT) model. Support for each node was tested using bootstrap method with 1000 replicates. Gene-specific primers were designed for amplifying conserved motif of each gene. PCR products were cloned into pMD 19-T vector (TaKaRa, Dalian, China) and confirmed by Sanger sequencing. Based on conserved motif sequences, gene-specific primers (Supplementary Table S1) were designed for conducting RACE-PCR to amplify target 5^′^and 3^′^cDNA ends, using SMARTer RACE cDNA amplification kit (Clontech, Mountain View, CA, USA). Amplification products of 5^′^and 3^′^ RACE were then cloned and sequenced to get full-length cDNAs. Four meiotic genes identified from *A. philoxeroides*,* ApASY1*, *ApMLH3*, *ApMPK4,* and *ApMMD1*, were also cloned and sequenced. They are responsible for homologous chromosome synapsis (Armstrong et al., 2002), crossover formation (Jackson et al., 2006), male-specific meiotic cytokinesis (Zeng et al., 2011) and general meiotic cell cycle progression (Yang et al., 2003b), respectively.

### Quantitative Real-Time PCR (qRT-PCR) Analysis

The expression patterns of B-class MADS-box genes and genes involved in meiosis were analyzed by quantitative real-time PCR (qRT-PCR). For B-class gene analysis, total RNAs were isolated from leaves, sepals, stamens, and carpels of normal and pistillate flowers, respectively. For meiotic gene analysis, total RNAs were isolated from the stamens at early developmental stages of normal and sterile flowers. The first-strand cDNA was made from 2 μg of total RNA using PrimeScript^TM^ RT Master Mix Perfect Real Time (TaKaRa, Dalian, China) following the manufacturer’s recommendations. The gene-specific primers used for qRT-PCR (Supplementary Table S1) were designed using PRIMERS3^5^. Real-time PCR was performed on a Roche LightCycler®2.0 machine (Roche diagnostics, Mannheim, Germany) using SYBR® Premix Ex TaqTM II (TliRNaseH Plus; TaKaRa, Dalian, China). The cycling parameters are as follows: initial denaturation (95°C for 30 s), 40 amplification cycles (95°C for 5 s and 60°C for 20 s), and followed by a melt cycle (60°C for 15 s). All reactions were run with three biological replicates and each with three technical replicates. *UBC10* was used as the reference gene to normalize the gene expression level. Quantification of the relative changes in gene expression was performed using the 2^-ΔΔ^*^C^*^T^ method (Livak and Schmittgen, 2001). Data represented three biological replicates with three technical replicates and were shown as average, with error bars representing standard deviations. Duncan’s test was used to determine the statistical significance of differences.

## Results

### *De novo* Transcriptome Assembly and Annotation

cDNA libraries were constructed and sequenced for the normal flower, male-sterile flower, and pistillate flower, respectively. A total of 107,160,189 raw reads accounting for 21.6 Gb of raw data were generated for the three libraries. After filtering, 104,487,087 clean reads (20.1 Gb) were retained and used for *de novo* assembly (**Table 1**). Each library was assembled independently, and then merged to generate the final assembly. After redundancy removal, a final set of 208,082 transcripts (≥200 bp) were obtained, with a mean length of 870 bp and N50 of 1,514 bp (**Table 2**). Of the transcripts retained, 169,183 (81.3%) transcripts were expressed in all three samples and 4.3% only in normal flowers.

**Table 1:** Summary of sequencing statistics.

	Normal flowers	Pistillate flowers	Male-sterile flowers	Total
Total raw reads	49,113,802	34,075,245	23,971,142	107,160,189
Total raw bases (bp)	9,920,988,004	6,883,199,490	4,842,170,684	21,646,358,178
Number of reads after trimming	48,046,265	33,347,238	23,093,584	104,487,087
Number of bases after trimming (bp)	9,286,674,933	6,463,064,464	4,432,813,071	20,182,552,468

**Table 2:** Summary of *de novo* assembly results.

	No
Total number of high quality assembled reads	104,487,087
Number of transcripts	208,082
Mean length (bp)	870
N50 (bp)	1,514
Longest transcript (bp)	18,515
Number of transcripts >5 Kb	663
Number of transcripts >10 Kb	23

A total of 83,878 (40.3%) transcripts were matched to 15,273 *A. thaliana* genes, covering 56.4% of *A. thaliana* genome. 3,980 (1.9%) transcripts were further identified by BLAST searches against the Phytozome database. Among the annotated transcripts, 56 were associated with A-, B-, C-, and E-class MADS-box genes (Supplementary Table S2), and 168 associated with 31 know meiotic genes responsible for homologous chromosome synapsis (Armstrong et al., 2002), male-specific meiotic cytokinesis (Zeng et al., 2011), general meiotic cell cycle progression (Yang et al., 2003b), and meiotic recombination (Osman et al., 2011), respectively (Supplementary Table S3).

### Detection of Differentially Expressed Genes

**FIGURE 2:**
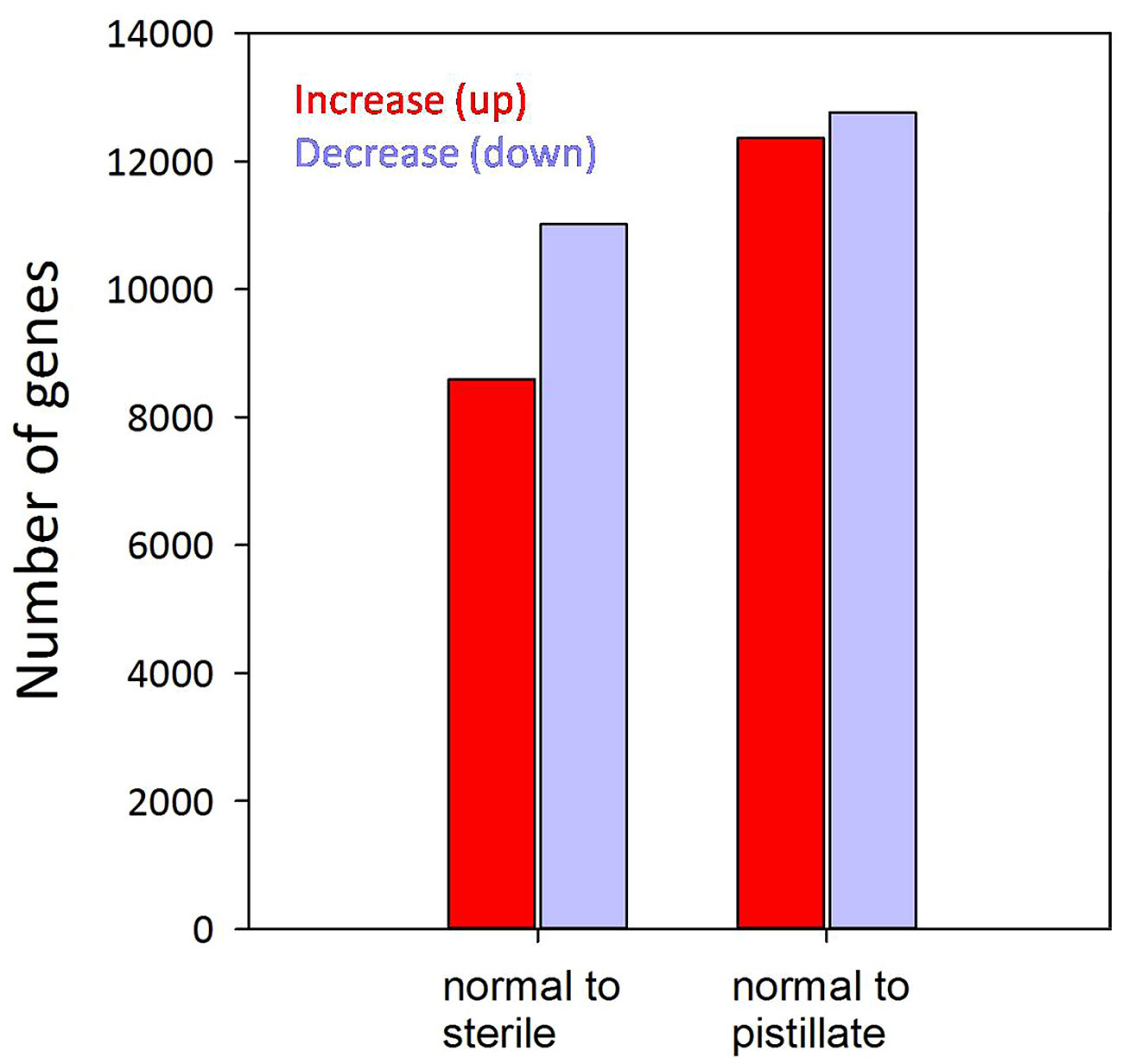
**Overview of differentially (FDR ≤0.001) expressed genes.** Red bars present the number of genes that increased expression while blue bars present the number of genes that decreased expression in the corresponding interval.

To identify molecular phenotypes associated with different patterns of anomalous stamen development in *A. philoxeroides*, expression patterns of annotated transcripts were compared between different types of flowers. Comparison between normal and pistillate flowers revealed 11,015 up-regulated and 8,591 down-regulated transcripts in the pistillate flower, using a FDR of 0.1% (**Figure 2**). 12,761 and 12,364 transcripts were up- and down-regulated, respectively, in the male-sterile flower compared to the normal flower. Transcripts associated with B-class MADS-box genes exhibited lower expression in the pistillate flower, while transcripts associated with A-, C-, and E-class genes showing no significant differences between normal and pistillate flowers (Supplementary Table S2). Transcripts associated with meiotic genes did not show significant decreases in male-sterile flowers compared with normal flowers, with the exception of transcripts associated with *AtMSH5* that showed decreased expression in the male-sterile flower (Supplementary Table S3).

**FIGURE 3:**
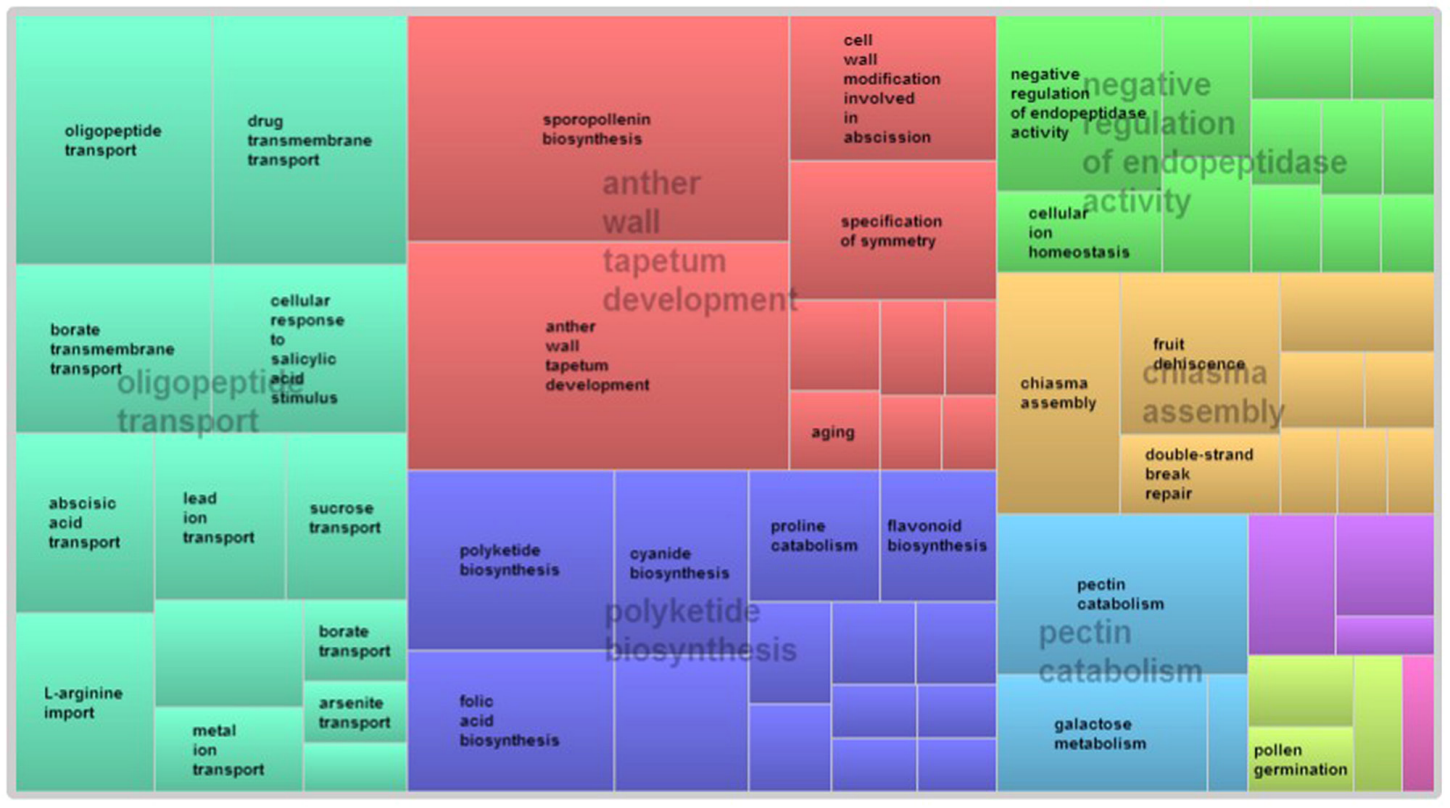
**REVIGO-summarized enriched GO terms among down-regulated genes in pistillated flowers.** Similar colors denote semantic similarity in the supercluster and the area of the rectangles is proportional to the significance of the over-representation of the GO term (-log10 *p*-value).

**FIGURE 4:**
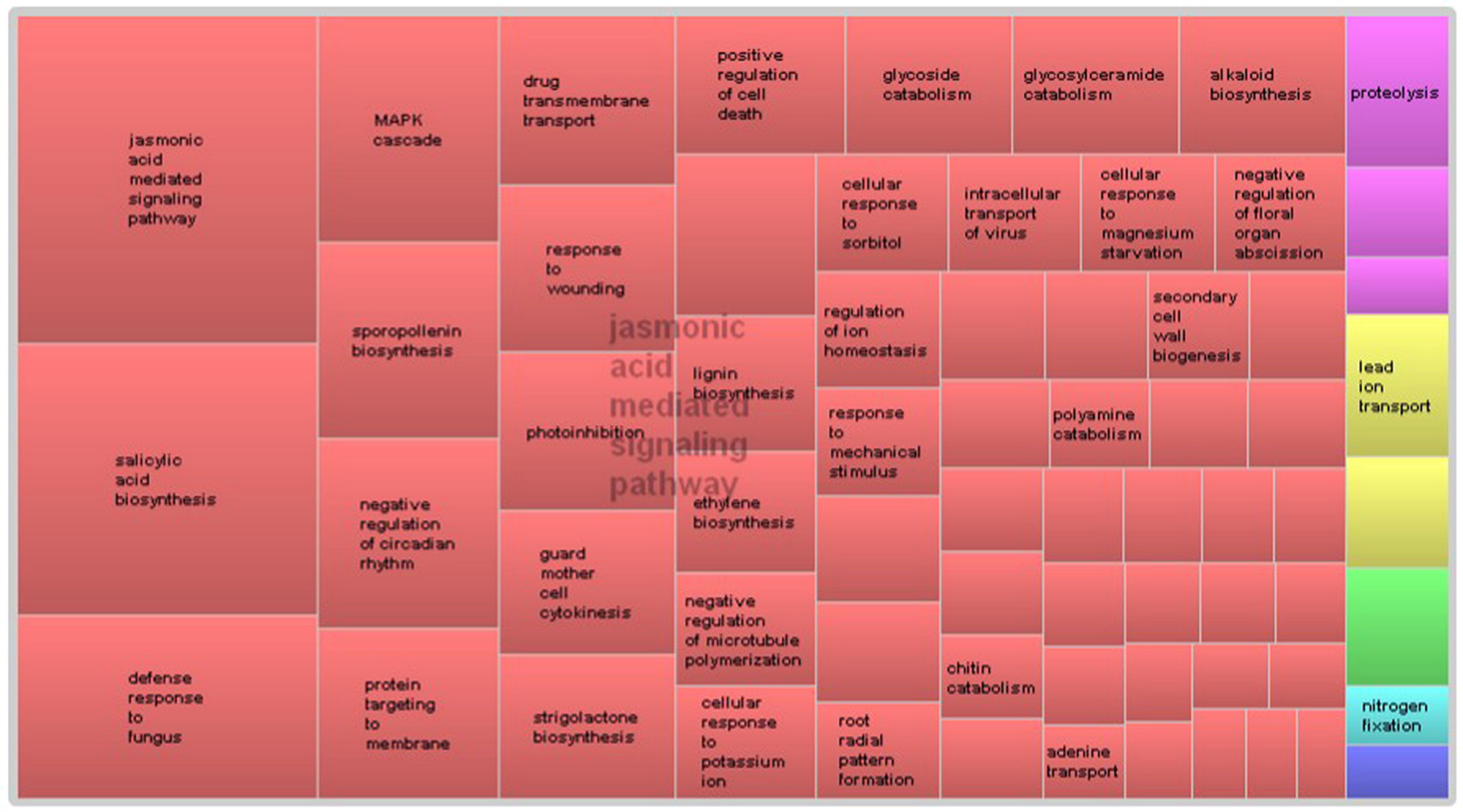
**REVIGO-summarized enriched GO terms among down-regulated genes in male-sterile flowers.** Similar colors denote semantic similarity in the supercluster and the area of the rectangles is proportional to the significance of the over-representation of the GO term (-log10 *p*-value).

GO term enrichment analysis of differentially expressed genes revealed additional enriched functional categories. Genes involved in polyketide biosynthesis, oligopeptide transport, anther wall tapetum development, pectin catabolism, and negative regulation of endopeptidase activity, showed decreased expressions in the pistillate flower (**Figure 3**). GO terms associated with the response to red or far red light, negative regulation of circadian rhythm, ATP-dependent chromatin remodeling and protein acetylation were also enriched in the down-regulated genes of the pistillate flower. In addition, GO term annotation highlighted that genes involved in the jasmonic acid (JA) mediated signaling pathway were strongly overrepresented among the differentially expressed genes between normal and male-sterile flowers (**Figure 4**), and most of these genes were expressed decreasely in the male-sterile flower. Genes involved in the biosynthesis of constituents required for pollen wall development and pollen maturation, such as sporopollenin, xanthophyll, cellulose, pectin, lipid, sugar, and various pollen proteins, were included in the supercluster of JA mediated signaling pathway (**Figure 4**).

### Validation the Expression of B-Function and Meiotic Genes in Anomalous Stamens

**FIGURE 5:**
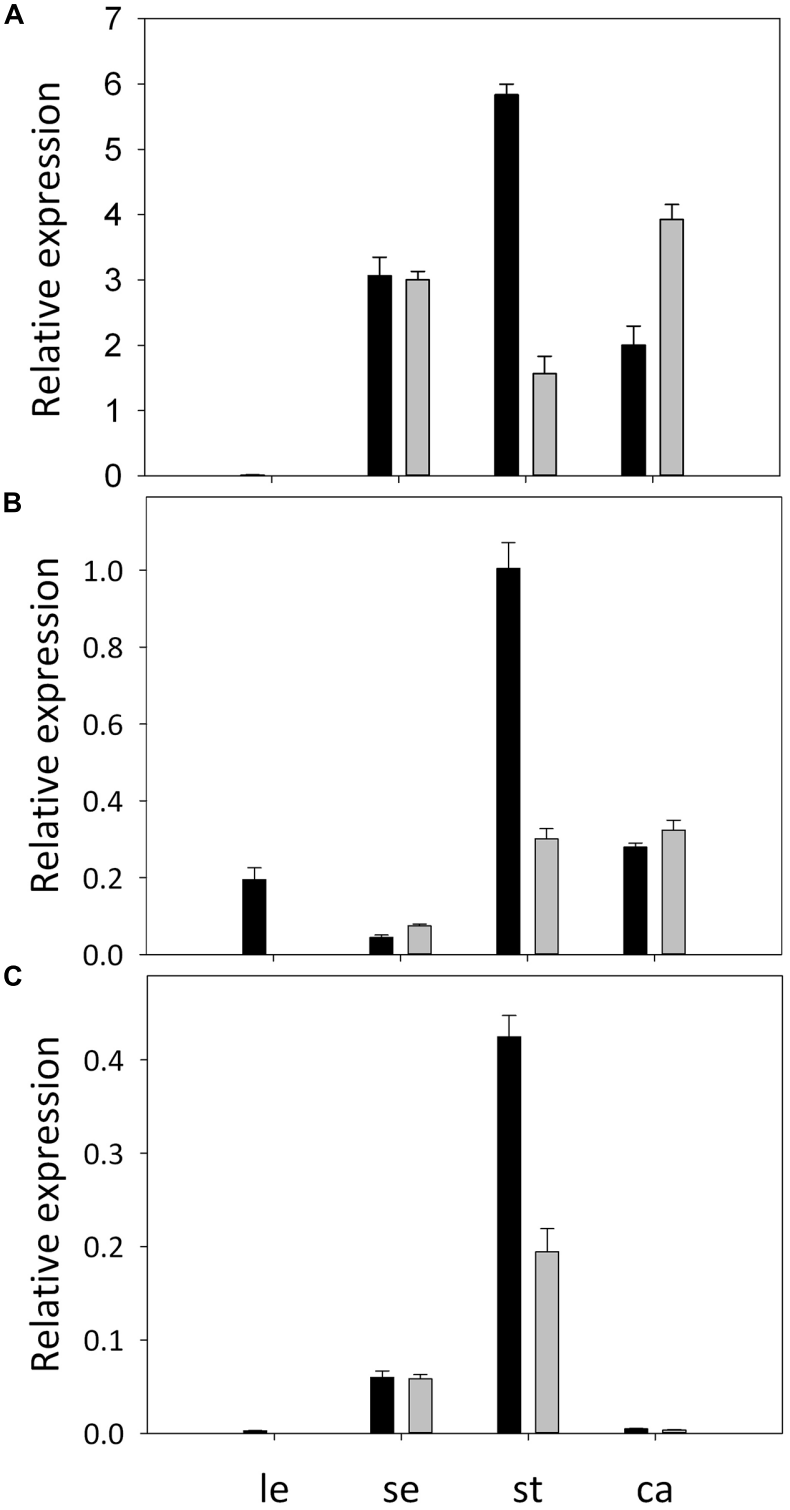
**Expression patterns of B-function genes in *A. philoxeroides*.** Relative expression of *ApAP3* **(A)**, *ApTM6* **(B)**, and *ApPI* **(C)** in tissues from normal flowers (black bars) and pistillate flowers (gray bars), as revealed by qRT-PCR. Tissues assayed are leaf (le), sepal (se), stamen (st), and carpel (ca). Error bars represent standard deviations of three replicates.

Full-length cDNAs of three B-class MADS-box genes were obtained from *A. philoxeroides*. They were clustered, respectively, with the *AP3*, *TM6,* and *PI* orthologs from other plant species in the phylogenetic tree (Supplementary Figure S1), and were thus designated, respectively, as *ApAP3*, *ApTM6,* and *ApPI*.* ApAP3* was 675 bp in length with an open reading frame corresponding to 224 deduced amino acid residues. *ApTM6* was 717 bp long, encoding a 238 amino acid protein, while *ApPI* containing a 654 bp open reading frame. The expression patterns of *ApAP3*, *ApTM6,* and *ApPI* in normal and pistillate flowers were validated by qRT-PCR. The results showed that three B-class genes were all expressed in the sepals of both flowers. However, the expression levels of *ApTM6* and *ApPI* were very low, and there were no significant differences in expression levels of three genes between two types of flowers (**Figure 5**). Three B-class genes were all highly expressed in the stamens of normal flowers, but the expression levels decreased by 73.2, 70.1, and 54.2%, respectively, in the stamens of pistillate flowers. Expressions of *ApAP3* and *ApTM6* were also detected in the carpels of both flowers but not for *ApPI*.

**FIGURE 6:**
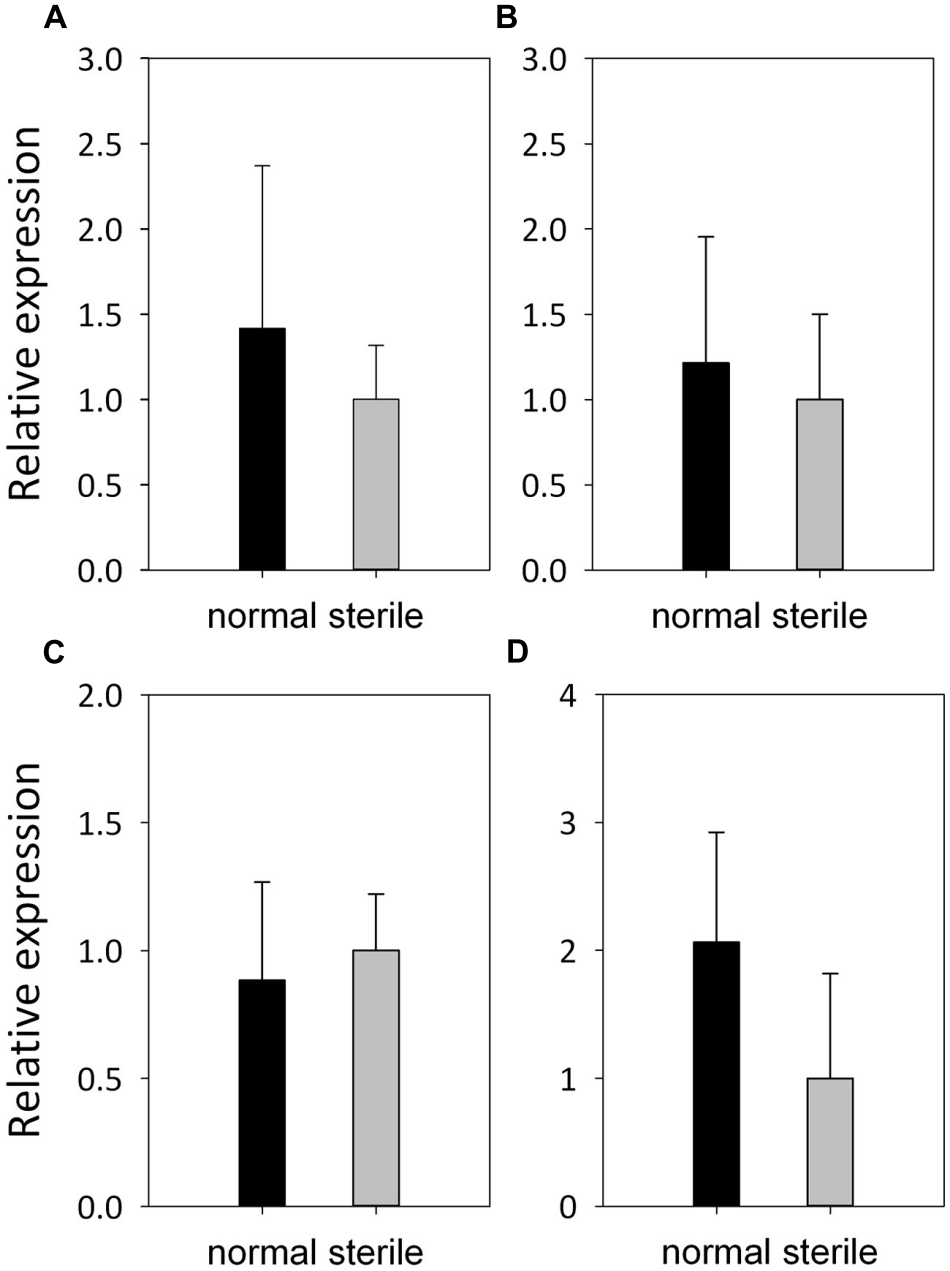
**Expression patterns of meiotic genes in *A. philoxeroides*.** Relative expression of *ApASY1* **(A)**, *ApMLH3* **(B)**, *ApMPK4* **(C)**, and *ApMMD1* **(D)** in anthers from normal flowers (black bars) and male-sterile flowers (gray bars), as revealed by qRT-PCR. Error bars represent standard deviations of three replicates.

The expression patterns of four meiotic genes,* ApASY1*, *ApMLH3*, *ApMPK4,* and *ApMMD1* involved in different processes of meiosis, were also validated by qRT-PCR. They were all expressed in the stamens of normal and male-sterile flowers, and did not show significant differences in expression levels between normal and male-sterile flowers (**Figure 6**).

## Discussion

Comparative transcriptome analysis revealed overall differences in gene expression between normal and anomalous flowers. Altered expressions of genes associated with stamen development were confirmed with qRT-PCR analyses. As revealed in other plants (de Martino et al., 2006), the expression levels of B-function MADS-box genes were significantly decreased in the stamens of *A. philoxeroides* pistillate flowers. B-function genes physically interact with C- and E-function genes to form quaternary complexes to specify stamen development (Airoldi, 2010). Because the expression levels of C- and E-function genes remained in *A. philoxeroides* pistillate flowers, the decreased expression of B-class genes was evidently responsible for the homeotic transformation of stamens into carpels in *A. philoxeroides*. Our results are in agreement with previous studies on homeotic variation in flowers. de Martino et al. (2006) showed that decreased expression of only one B-class gene could result in a complete transformation of the stamens into carpel-like organs in tomato. The deficiency of B-class MADS-box genes also caused homeotic conversions of stamens into carpels in *Arabidopsis* (Jack et al., 1992; Goto and Meyerowitz, 1994), *Antirrhinum* (Schwarz-Sommer et al., 1992; Tröbner et al., 1992), tomato (Rasmussen and Green, 1993; Olimpieri and Mazzucato, 2008) and wheat (Hama et al., 2004; Yamada et al., 2009). The reduced expression of B-function genes in *A. philoxeroides* seems not to result from the loss-of-function mutation in B-class genes because, by cloning and sequencing *ApAP3*, *ApTM6,* and *ApPI* from different plants, we did not find significant sequence variation between normal and pistillate flowers. Deng et al. (2011) and Liu et al. (2011) showed that environmental variation, especially soil nutrient heterogeneity, can induce floral gender transformation in *A. philoxeroides*, with the stamens of monoclinous flowers being completely or partially transformed into carpels (Deng et al., 2011; Liu et al., 2011). It is unclear, however, by which mechanisms the change in environment is sensed, transduced, and finally elicits modifications to the selective expression of B-class genes in different habitats. In addition to B-class genes, transcriptome analysis also revealed other genes that were differentially expressed between normal and pistillate flowers, and were enriched for a wide range of molecular function categories. The differential expression of genes involved in GA signaling and epigenetic regulation is of special interest. It has been revealed that floral homeotic genes (*AP3* and *PI*) were targets of GA signaling in flower development (Yu et al., 2004). GA probably promoted stamen development by upregulating expression of the floral meristem identity gene *LEAFY* (*LFY*), which in turn upregulates expression of the B-class MADS-box gene *AP3* (Plackett et al., 2011). Reduction in GA synthesis might lead to a reduced expression of *AP3*, and thereby produces abnormal flowers with carpelloid stamens (Kamata et al., 2013). Studies on the *stamenless* mutant also showed evidences that stamen identity in tomato depended on gene–hormone interactions (Quinet et al., 2014). Additionally, it has been shown that epigenetically regulated ectopic expression of flower homeotic genes may alter floral organ identity (Kapoor et al., 2005; Pu et al., 2013). Histone modification and ATP-dependent chromatin remodeling are also involved in the regulation of spatiotemporal-specific expression of genes that lead to patterning, specification, and morphogenesis of flowers (Gan et al., 2013). Mutation in the chromatin-remodeling ATPases BRAHMA led to the occurrence of carpelloid structures in the third whorl of *Arabidopsis* flowers (Hurtado et al., 2006; Wu et al., 2012). It has been suggested that MADS-domain proteins may closely interact with chromatin remodeling factors to facilitate chromatin opening and transcription initiation (Smaczniak et al., 2012; Guo et al., 2015).

Most of the meiotic genes investigated in this study were normally expressed in the male-sterile flower of *A. philoxeroides*. This result was inconsistent with our original hypothesis. The cytotype of *A. philoxeroides* found in China is a hexaploid, with approximately 100 chromosomes. Abnormal male meiosis is often used as a cytological explanation for pollen sterility in polyploidy plants. The most common meiotic abnormalities were those related to irregular chromosome segregation due to polyploidy, leading to the formation of chromosomally imbalanced gametes and aneuploidy. To date, little cytogenetic work has been done on the meiotic process of the invasive *A. philoxeroides*, due to the small size and apparent similarity of the chromosomes. It is unclear whether meiosis proceeds normally in the *A. philoxeroides* male-sterile flower. The normal expression of meiotic genes in the male-sterile flower seems to suggest that the meiotic abnormality is unlikely responsible for the pollen sterility observed in *A. philoxeroides*, or segregation defects sometimes occur during meiosis II after meiosis I has proceeded normally. Other anther developmental defects may also generate male-sterile phenotypes (Sanders et al., 1999; Sakata and Higashitani, 2008). In consistent with this prediction, we found that many genes involved in the JA mediated signaling pathway were strongly down-regulated in the male-sterile flower. JA is critical for late stages of stamen development, regulating filament elongation, anther opening, and pollen maturation. (Turner et al., 2002; Song et al., 2013; Wasternack and Hause, 2013).* Arabidopsis* mutants impaired in JA biosynthesis exhibited non-viable pollen and delayed anther dehiscence (Wasternack and Hause, 2013). JA signaling also played crucial roles in a variety of biosynthetic pathways for the components of pollen intine and exine, and various storage materials accumulated during pollen maturation (Mandaokar et al., 2003, 2006; Wasternack and Hause, 2013). As a result of defects in the JA signaling pathway, a lot of genes involved in the biosynthesis of constituents required for pollen wall development and pollen maturation were also down-regulated in the male-sterile flower of *A. philoxeroides*. Thus, defects in JA synthesis and/or JA signaling, as well as subsequent physiological disorders, might be potential causes for male sterility in *A. philoxeroides*.

Overall, the invasive *A. philoxeroides* exhibited a high level of plasticity in stamen development. This high level of plasticity is clearly resulted from relaxed selective constraints on sexual reproduction. After being introduced into China, *A. philoxeroides* spreads mainly by vegetative (clonal) propagules, though it retains the principal ability to reproduce both sexually and asexually in its native range. Although genetic factors, such as changes in ploidy, may play a role in causing reduced sexual fertility, the shift toward asexual reproduction is more likely promoted by biotic and/or abiotic limiting factors of the environment in exotic *A. philoxeroides* populations. Clonal reproduction probably helps the plants of *A. philoxeroides* to overcome the negative effects associated with low population densities during colonization and enhances exploitation of ubiquitous environmental heterogeneity, facilitating range expansion. Asexual reproduction is particularly common among introduced species (Kronauer et al., 2012), and shifts from sexual to asexual reproduction in the exotic range have been observed in several clonal invaders (Sculthorpe, 1967; Ornduff, 1987; Hollingsworth and Bailey, 2000). Repeated cycles of colonization and low-density may favor uniparental reproduction because selfing and asexuality provide plants with reproductive assurance (Eckert et al., 2006; Barrett et al., 2008). Meanwhile, genetic sterility may be induced by environmental suppression of sexual recruitment because natural selection no longer strongly maintains the traits involved in sex (Eckert, 2002). As a result, ‘neutral’ sterility mutations and developmental abnormalities accumulate in highly clonal populations, as shown in exotic *A. philoxeroides* plants. Thus, the occurrence of various types of stamen abnormalities could be explained by the hypothesis that sex were degraded for they no longer increase fitness (Larkin et al., 2007; Tamura et al., 2013). Sexual sterility may be first induced by ecological factors, the resulting genetic sterility may, in turn, further hamper sexual recruitment in clonal populations, facilitating the evolution of asexual reproduction in clonal plants.

## Author Contributions

ZZ collaborated in the design of the research, collected plant materials, prepared RNA samples for high-throughput sequencing, performed the bioinformatics analyses, interpreted the results and wrote the first draft of the manuscript. CZ helped in the design of the experiments as well as RNA samples preparation, performed the experiments with B-class MADS-box genes, including plant materials collection, cDNA full-length cloning, phylogenetic analysis and qRT-PCR. Moreover, she made graphs and wrote the draft about B-class MADS-box genes. JY made substantial contributions to the design of the research, analysis of next-generation sequencing data and manuscript revision. All authors have read the final version of the manuscript and agree with its content.

## Conflict of Interest Statement

The authors declare that the research was conducted in the absence of any commercial or financial relationships that could be construed as a potential conflict of interest.

## Footnotes

1 http://www.arabidopsis.org/

2 http://www.phytozome.net/

3 http://revigo.irb.hr/

4 http://www.ncbi.nlm.nih.gov/

5 http://bioinfo.ut.ee/primer3/
