# Supplementary material for: Molecular phenotypes associated with anomalous stamen development in Alternanthera philoxeroides
Source: Front Plant Sci. 2015 Apr 14;6:242. doi: 10.3389/fpls.2015.00242 (PMC4396347; doi:10.3389/fpls.2015.00242)
Supplement: Supplementary file 1 [file Data_Sheet_1.ZIP › data sheet 1/Figure S1.pdf]

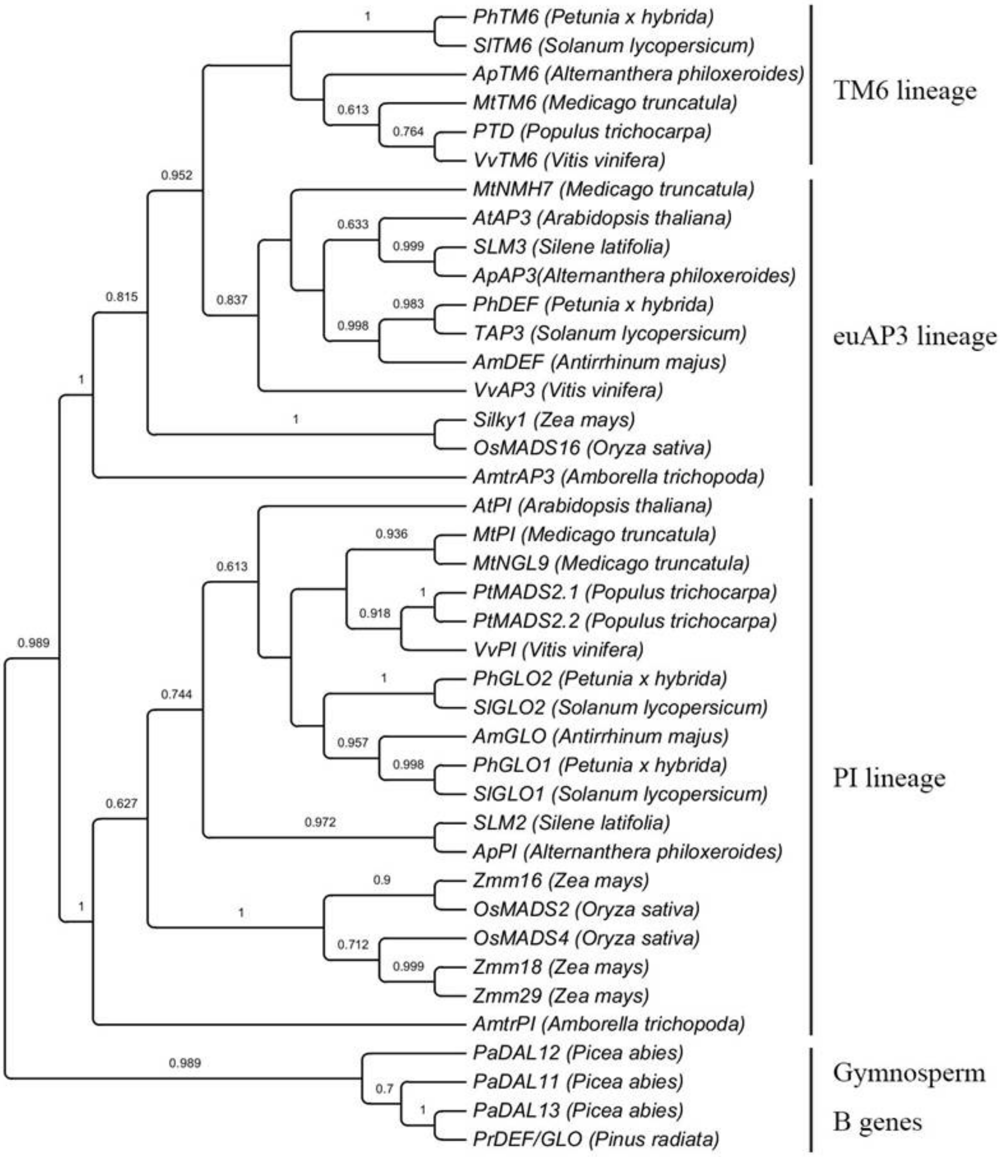

Supplementary Figure S1 Neighbor-joining tree of B-function MADS-box genes from a selection of diverse species.

The representative MADS-box genes for major subfamilies are indicated on the right side of the tree. Bootstrap values exceeding 50% are showed along the branches. Paleo*AP3*/*DEF* homologs from gymnosperms was used as an outgroup.
