# Supplementary material for: Molecular phenotypes associated with anomalous stamen development in Alternanthera philoxeroides
Source: Front Plant Sci. 2015 Apr 14;6:242. doi: 10.3389/fpls.2015.00242 (PMC4396347; doi:10.3389/fpls.2015.00242)
Supplement: Supplementary file 1 [file Data_Sheet_1.ZIP › data sheet 1/Table S1.pdf]

Supplementary Table S1 Primer sequences

Purpose	Gene name	Primer sequence (5'-3')	Primer sequence (5'-3')
**Conserved domain cloning**		**Forward primer**	**Reverse primer**
	AlAP3	AGTTACGCATGATCCTGTTC	GGTATCACGACAAAGGAGGT
	AlTM6	ACAAGAAGTCAGTGATGCCA	CTTCTTACACGAACACAGCA
	AlPI	GGAAGAGGAAAGATTGAGATTA	TATCCTCAAGTCATCATTTTCC
**Cloning by RACE**		**GSP1 (for 5' RACE)**	**GSP2 (for 3' RACE)**
	AlAP3	GGCTGCAACCTCAAAGCAAGTATCCGA	GGCGAAGGATGGGAGATTGTTTGGA
	AlTM6	TGGCATCCCAAATTCTCTTCCCTGAG	ATGCTTATCATACCCCTTCGTCCTCGG
	AlPI	GGGCACATTCAAGCAAGCCGCAAGT	TTGCTATGAGCCGCCAACCTCGCCT
**CDS cloning**		**Forward primer**	**Reverse primer**
	AlAP3	ATGGCAAGAGGAAAAATTCA	TTCAGTATCTGCTAACAAGCAC
	AlTM6	ATGTTAAATAACGTAAAAATGGGG	TCAAGCAAGCCGCAAGTCATG
	AlPI	ATGGGAAGAGGAAAGATTGAG	TTAAACCGTATCCTGCAAGTTG

qRT-PCR		Forward primer	Reverse primer
	AlAP3	TTCTTAGCCCTGGTATCACGAC	CCAAACAATCACCCATCCTTC
	AlTM6	TTAGAAACCAACAAGAGGCTGC	AACCCTGGCATCACTGACTTC
	AlPI	GCACAAGCAAGGAATGATGG	TGTGGCATTGTTGATAGTCTCC
	AlUBC10	AGCCCTGCTTTAACCATTTCC	ATACTTCTGGGTCCAGCTCCTG
