# Supplementary material for: Molecular phenotypes associated with anomalous stamen development in Alternanthera philoxeroides
Source: Front Plant Sci. 2015 Apr 14;6:242. doi: 10.3389/fpls.2015.00242 (PMC4396347; doi:10.3389/fpls.2015.00242)
Supplement: Supplementary file 1 [file Data_Sheet_1.ZIP › data sheet 1/Table S2.pdf]

Supplementary Table S2 Putative genes in *A. philoxeroides* corresponding to *A. thaliana* floral homeotic genes.

Gene name	Best hit	Length	E-value	Annotation source	Log_2 (Fold Change)
AtAP1	s7_comp61300_c0_seq1	1214	1E-98	AT1G69120	**
*AtAP*2	s7_comp68963_c0_seq6	1979	9E-102	AT4G36920	**
*AtAP*3	s7_comp63221_c0_seq7	1271	2E-76	AT3G54340	1.19
AtPI	s9_comp46392_c0_seq1	859	1E-68	AT5G20240	4.50
AtAG	Contig9617	1638	6E-97	AT4G18960	-0.49
AtSEP1	s7_comp63454_c0_seq2	1236	2E-116	AT5G15800	-0.55
AtSEP3	Contig8774	1245	6E-104	AT1G24260	0.10

** Not significant when FDR$\leq$ 0.001. Log_2 (Fold Change) is the log2 ratio of gene transcript between normal flowers to pistillate flowers for floral homeotic genes. If log_2 (Fold Change)>1, the value indicates transcript is abundant in normal flowers. If log_2 (Fold Change)<-1, the value indicates transcript is abundant in pistillate flowers.
