# Supplementary material for: Molecular phenotypes associated with anomalous stamen development in Alternanthera philoxeroides
Source: Front Plant Sci. 2015 Apr 14;6:242. doi: 10.3389/fpls.2015.00242 (PMC4396347; doi:10.3389/fpls.2015.00242)
Supplement: Supplementary file 1 [file Data_Sheet_1.ZIP › data sheet 1/Table S3.pdf]

Supplementary Table S3 Putative genes in *A. philoxeroides* corresponding to *A. thaliana* meiotic genes.

Gene name	Best hit	Length	E-value	Annotation source	Log_2 (Fold Change)
**DNA double-strand break formation**					
AtSPO11-1	s9_comp46275_c0_seq1	1548	3E-152	AT3G13170	-2.74
AtSPO11-2	Contig9912	1462	1E-170	AT1G63990	**
AtPRD1	s7_comp10707_c0_seq1	1247	1E-73	AT4G14180	**
AtPRD2	Contig39864	1459	2E-76	AT5G57880	-1.67
AtPRD3	s8_comp49307_c0_seq1	1739	1E-43	AT1G01690	-1.33
**Processing of DNA double-strand breaks**					
AtMRE11	Contig11705	2758	0	AT5G54260	**
AtRAD50	Contig42720	4632	0	AT2G31970	**
AtNBS1	Contig15306	2276	1E-96	AT3G02680	-0.53
AtCOM1	Contig21843	1444	5E-66	AT3G52115	**
**DNA strand exchange: RecA homologues and accessory factors**					
AtRAD51	Contig7829	1554	0	AT5G20850	-0.52
AtDMC1	Contig7193	1455	0	AT3G22880	-0.62
AtRAD51C	Contig6927	1462	8E-154	AT2G45280	**
AtXRCC3	Contig1778	606	2E-53	AT5G57450	**
AtRPA1a	Contig14711	2375	0	AT2G06510	-0.70
AtBRCA2	Contig17990	1225	9E-34	AT4G00020	**
AtMND1	Contig25481	999	2E-99	AT4G29170	-1.99

AtAHP2(AtHOP2)	Contig40848	1090	4E-107	AT1G13330	-0.72
**Proteins required for crossover formation**					
AtMER3/RCK	s7_comp70574_c0_seq35	3902	0	AT3G27730	**
AtMSH4	Contig38919	2705	0	AT4G17380	**
AtMSH5	Contig11068	1974	0	AT3G20475	2.08
AtHEI10(AtZIP3)	Contig10439	1345	7E-155	AT1G53490	**
AtSPO22(AtZIP4)	Contig31655	4410	0	AT5G48390	-1.48
AtPTD	s7_comp60202_c0_seq2	1246	7E-97	AT1G12790	**
AtSHOC1	Contig28711	3868	9E-112	AT5G52290	-1.14
AtZYP1a	Contig16758	3128	0	AT1G22260	-0.74
AtZYP1b	s9_comp6542_c0_seq1	231	7E-14	AT1G22275	**
AtMLH3	Contig17089	4330	1E-102	AT4G35520	-0.50
AtMUS81	Contig13947	2242	4E-104	AT4G30870	**
**Homologous chromosome synapsis**					
AtASY1	s7_comp66999_c0_seq1	2780	0	AT1G67370	0.64
**Male-specific meiotic cytokinesis**					
AtMPK4	Contig46544	1709	0	AT4G01370	**
**General meiotic cell cycle progression**					
AtMMD1	Contig10814	2209	0	AT1G66170	**

** Not significant when FDR≤ 0.001. Log_2 (Fold Change) is the log2 ratio of gene transcript between normal flowers to male-sterile flowers for meiotic genes. If log_2 (Fold Change)>1, the value indicates transcript is abundant in normal flowers. If log_2 (Fold Change)<-1, the value indicates transcript is abundant in male-sterile flowers.
